# GPS suggests low physical activity in urban Hispanic school children: a proof of concept study

**DOI:** 10.1186/1687-9856-2014-25

**Published:** 2014-12-15

**Authors:** Aaron L Carrel, Jeffrey S Sledge, Stephen J Ventura, Jens C Eickhoff, David B Allen

**Affiliations:** Department of Pediatrics, University of Wisconsin, 600 Highland Avenue, H4-436, Madison, WI 53792-4108 USA; Land Information and Computer Graphics Facility, University of Wisconsin, 600 Highland Avenue, H4-436, Madison, WI 53792-4108 USA

**Keywords:** Hispanic, GPS, School assessment, Exercise, School, Child

## Abstract

**Background:**

Urban environments can increase risk for development of obesity, insulin resistance (IR), and type 2 diabetes mellitus (T2DM) by limiting physical activity. This study examined, in a cohort of urban Hispanic youth, the relationship between daily physical activity (PA) measured by GPS, insulin resistance and cardiovascular fitness.

**Methods:**

Hispanic middle school children (n = 141) were assessed for body mass index (BMI), IR (homeostasis model [HOMA-IR]), cardiovascular fitness (progressive aerobic cardiovascular endurance run [PACER]). PA was measured (GPS-PA) and energy expenditure estimated (GPS-EE) utilizing a global positioning mapping device worn for up to 7 days.

**Results:**

Students (mean age 12.7 ± 1.2 years, 52% female) spent 98% of waking time in sedentary activities, 1.7% in moderate intensity PA, and 0.3% in vigorous intensity. GPS analysis revealed extremely low amounts of physical movement during waking hours. The degree of low PA confounded correlation analysis with PACER or HOMA-IR.

**Conclusions:**

Levels of moderate and vigorous intensity PA, measured by GPS, were extremely low in these urban Hispanic youth, possibly contributing to high rates of obesity and IR. Physical movement patterns suggest barriers to PA in play options near home, transportation to school, and in school recess time. GPS technology can objectively and accurately evaluate initiatives designed to reduce obesity and its morbidities by increasing PA.

## Background

Built environments can impede, or encourage active lifestyles for children [1, 2]. Social environments influence how children choose, or are permitted, to interact with the environment. Reduced daily physical movement is one factor contributing to overweight/obesity, now the most common medical condition of childhood in the US. Certain ethnic minority populations, including Hispanic children, are at greater risk for obesity and its related morbidities [3–5]. Increasing numbers of children also fail to meet minimum recommendations for physical activity [6]. Both poor physical fitness and obesity are risk factors for T2DM and cardiovascular disease [7–12]. In fact, cardiovascular fitness (CVF) is a stronger predictor of mortality than obesity [13, 14]. Increased physical activity and fitness in children is associated with reduced risk for diabetes and other improved health outcomes. Thus, identifying and altering modifiable barriers to physical activity and lifestyle behaviors during childhood is paramount [15–17]. Successful public health interventions often utilize the Social Ecological Model (SEM) to address interacting environments at the individual, home, school, community, and society levels [18, 19]. GPS offers the technology to document where, when, and how much activity is taking place.

One particularly attractive target for assessment and potential improvement of physical activity for children and adolescents is the school-day routine: travel to/from, recess movement, and after-school activities [16, 17]. Since school attendance is an experience shared by the vast majority of children, the school environment and routine can potentially address low levels of physical activity (PA) and higher levels of sedentary behaviors, both of which are associated with IR. Most studies of childhood PA have relied on recall or physical activity logs, and very little objectively measured data is available regarding physical activity patterns of minority youth, including Hispanic children, who have a high rate of obesity, T2DM, and low fitness [20]. In this study, we utilize GPS to measure daily physical activity outside of classroom time in urban Hispanic youth and its relationship to BMI, cardiovascular fitness, and insulin resistance.

## Methods

Children (n = 141) from the Bruce-Guadalupe middle school (grades 5–8) in Milwaukee, Wisconsin, were invited to participate. Human Subjects Committee at the University of Wisconsin approved all procedures, and informed written consent was obtained from student and parent before study enrollment. All consenting was done with both Spanish and English speaking investigators to allow families the best opportunity to ask questions. All students were Hispanic (78% Mexican and 21% Puerto Rican) children. Students had a mean age of 12.7 (±1.2), and 52% were females. Students underwent assessment of cardiovascular fitness measured by the progressive aerobic cardiovascular endurance run (PACER), calculation of body mass index (BMI), and a subset (n = 55) underwent fasting blood work performed for insulin and glucose (HOMA-IR) after a 12 hour fast.

Children wore Global Positioning (GPS) receivers (QStarz 1300S carried in pocket, backpack or worn on a lanyard) to track and record daily physical and sedentary activity. GPS receivers with unique unit identifiers were assigned to students, who were asked to keep the GPS unit on their person and wear at all times outside of school classroom time, with the exception of sleeping and showering for a 7-day time period. The intent was to capture activity before or after school, or on weekends, rather than “in classroom” time. Each receiver was cleared and set to record time and position at a one second intervals using WAAS enabled satellite signal detection. Theoretical position resolution at this standard was less than three meters.

School staff received training, software, and support for performing Fitnessgram® testing including PACER and BMI determination at the school. Staff was trained to download and store data and to recharge and clear GPS units. Installation of GPS download and viewer software was accomplished by installation in the school’s information technology system.

PA was assessed using a geospatial model to equate GPS-recorded movement with energy expenditure. Using ArcGIS 9.X software, a community level “map” of movement by type (e.g., walking, running, motorized transport) was created to predict children’s energy expenditure (EE; in kilojoules) as they move through the community. For each child, energy expenditures were predicted from position (e.g., GPS) and movement, producing highly accurate records of spatio-temporally placed EE. GPS recordings were considered evaluable for analysis if the GPS unit was active for at least 90 minutes on a given day. In order to determine the daily average over the 7-day recording period for each participant, the mean values for each GPS measure (energy expenditure, distribution, minutes and percent time spent on activity) across the recording period were calculated and analyzed.

Levels of EE were defined by velocity as recorded by the GPS units. The units automatically stopped recording when motionless for extended periods, thus only time spent in activity was included in “GPS active time.” GPS data were processed into motion tracks in ArcGIS 9.x, using a standard spline function over three second intervals to smooth data spikes. Mode of travel was distinguished by acceleration signature coupled with peak and or sustained velocity. Time segments were manually interpreted from tracks and entered into a database for subsequent analysis.

Sedentary was defined as either lack of significant motion, or motion at rates and track patterns indicating travel in a motorized vehicle. Lack of motion was defined as less than 0.45 m/s (about 1 mile/per hour). Moderate intensity of activity was between 0.45 and 1.35 m/s, and vigorous activity was greater than 1.35 m/s in track patterns that did not correspond to vehicular travel. These distinctions are based on accepted definitions of NHANES datasets. Additionally, these GPS units included accelerometer triggers that helped us distinguish between the unit being at rest and unused, as opposed to being worn but with no positional change.

Cardiovascular fitness (CVF) was assessed using the PACER test, in which subjects run back and forth along a 20-meter shuttle run, and each minute the pace required to run the 20 meters quickens. The pace is set from a pre-recorded audio file or CD. The initial running speed is 8.5 km/hour, and the speed increases by 0.5 km/hour every minute. The test is finished when the subject fails to complete the 20-meter run in the allotted time twice. The PACER is expressed as number of laps completed [21]. PACER Z-score were calculated based upon Wisconsin references [22]. A single teacher performed all PACER testing after undergoing certified training in PACER testing procedures.

A 5 ml fasting blood sample was obtained for insulin and glucose levels on a single 12 hour fasting blood specimen. Glucose was determined by hexokinase method and insulin by chemiluminescent immunoassay (University of Wisconsin Hospitals and Clinics Laboratory, Madison, WI). HOMA-IR was calculated from glucose and insulin values (fasting glucose (mg/dL) × fasting insulin (μU/ml)/405).

All analyses were performed using SAS software version 9.2 (SAS Institute, NC). Demographic variables were summarized in terms of means ± standard deviations or as percentages. The distribution of GPS measures was highly skewed so that medians and ranges were used to summarize these measures. In order to account for the daily variability in the GPS measures across the 7 days recording period, the analyses of GPS measures were weighted using the inverse of the standard errors of the estimated mean GPS values as weights. Nonparametric, partial Spearman’s rank correlation coefficients were calculated to evaluate the associations between each GPS measures and BMI, fitness and insulin resistance measures. Since there was a large variation in the time for which the GPS device was active, the correlation analysis was adjusted by the median daily time the GPS device was active. Fisher’s z-transformation was used to construct 95% confidence intervals for the correlation coefficients. A two-sided 5% significance level was used for all statistical tests.

## Results

Complete data was collected on 141 students. A subset of 55 underwent fasting blood work; no differences were found comparing the group obtaining blood work with respect to mean BMI z-score, HOMA-IR, or PACER z-score. Mean BMI z-score was 0.91 (±1.0), mean HOMA-IR was 4.2 (±2.9), and mean PACER z-score was 0.31 (±0.98), based on age and gender (Table 1). Forty-nine percent of students had a BMI >85^th^-94^th^ percentile (overweight), and 29% of students had a BMI >95^th^ percentile (obese). PACER z-score was negatively correlated with BMI z-score (r = -0.47, 95% CI: -0.58 – -0.33; p < 0.00001) and with HOMA-IR (-0.60, 95% CI: -0.70 – -0.48; (p < 0.0001).

**Table 1:** **Demographics of study population**

	N	Mean	SD
Age (yrs)	141	12.7	1.2
BMI	141	23.0	5.1
BMI z-score	141	0.91	1.03
PACER (laps)	124	39.7	20.2
PACER z-score	122	0.31	0.98
Insulin	55	20.0	15.7
Glucose	55	89.4	6.7
HOMA IR	55	4.2	2.9
		N	%
Gender	141		
Male		73	48%
Female		68	52%
Overweight (BMI percentile ≥85^th^)	136	66	49%
Obese (BMI percentile ≥95^th^)		39	29%

There was a total of 519 days with evaluable (at least 90 minutes of active recording) GPS measurements across the 7-day period. The mean number of days with evaluable GPS measurements per student was 3.6 (±1.6).

**Figure 1:**
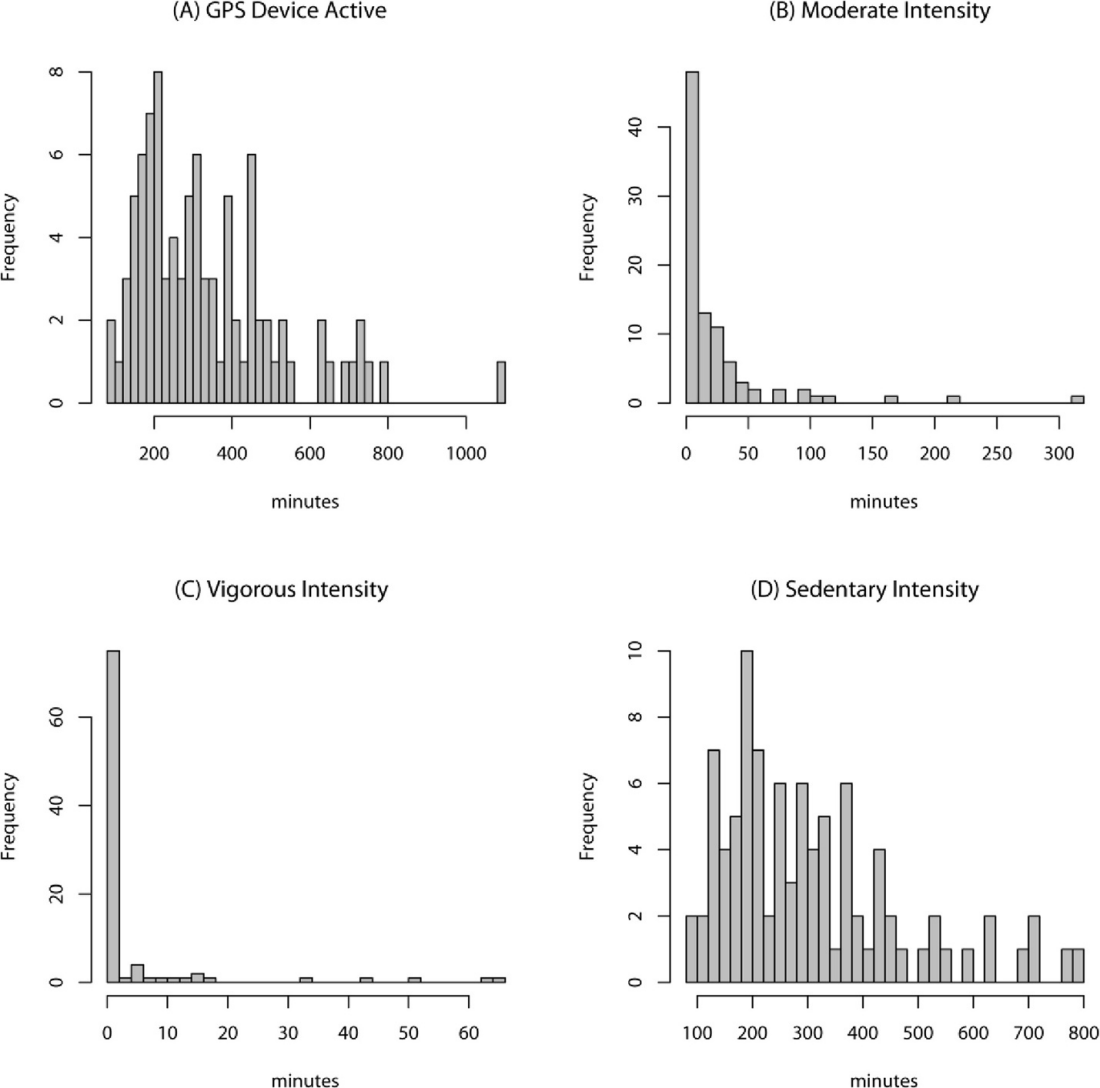
**Distribution of daily average GPS time measures: (A) GPS device activated; (B) moderate intensity activity; (C) vigorous intensity activity; (D) sedentary intensity activity.**

The GPS device was activated daily with a median duration of 310 minutes (range 97 – 1086). Students spent a median time of 6 minutes (range 1 – 318 minutes) on moderate intensity PA, 0 minute (range 0 – 65 minutes) on vigorous intensity PA and 294 minutes (range 94 – 780 minutes) on sedentary activities (Table 2, and Figure 1). The median daily time spent in a motorized state was 18 minutes (range 0 – 316 minutes). Seventy seven percent of participants spent ≤10% of waking time on moderate intensity activities, 90% spent <5% of the time on vigorous intensity activities, and 77% spent no time in vigorous intensity activity (Figure 1). Forty-five percent of the participants spent at a daily average of least 20 minutes in a motorized vehicle.

**Table 2:** **Summary statistics of daily average GPS measures**

	Median ^†^	Range
GPS active time (minutes)	310	97 – 1086
Time (minutes)		
Moderate intensity	6	1 – 318
Vigorous intensity	0	0 – 65
Sedentary	294	94 – 780
% Time spent		
Moderate intensity	2	0 – 42
Vigorous intensity	0	0 – 16
Sedentary	97	58 – 100
Energy (kcal expended)		
Moderate intensity	19	0 – 559
Vigorous intensity	0	0 – 205
Distribution		
Moderate intensity	1	0 – 31
Vigorous intensity	0	0 – 10

^†^Weighted using the inverse of the standard error of the estimated mean value across the 7-day study period.

## Discussion

Daily physical activity, objectively measured by GPS, was extremely low in this school-based cohort of urban Hispanic youth. Most subjects engaged in almost no vigorous activity during the study period, only 22% spent >10% of waking time (~90 minutes) in moderate activity, and the median percentage time spent in sedentary state was 98%. Median energy expenditure attributed to moderate and vigorous PA was 18 and 0 calories/day, respectively. GPS analysis revealed extremely low amounts of physical movement. The degree of low PA confounded correlation analysis with PACER or HOMA-IR. High levels of time were spent in motorized transport and low amounts of PA occurred both at the school and home environment. Since current national guidelines recommend a daily total of 60 minutes of moderate-to-vigorous activity (MVPA) for children [23], these data indicate that for many urban youth, there is a large gap between such recommendations and the realities of their daily life.

To evaluate the impact of public health interventions designed to increase physical activity, reliable measurements of movement are essential. This study demonstrates that GPS can provide reliable and objective measures of duration, distance, and intensity of physical movement. In addition, specific GPS-generated time-activity patterns can suggest barriers to movement and targeted interventions to address them. Students generally viewed using GPS very positively, and use of GPS enabled analysis of their specific physical activity behaviors and transportation choices and routes. This study provides proof-of-concept data that GPS tracking can be an effective research tool to accurately document duration of physical activity, intensity of physical activity, and specific location of physical activity [23]. For future intervention studies, linking GPS data to metabolic measurements in children provides an opportunity to objectively and accurately evaluate the impact of physical activity changes on metabolic health.

Factors influencing children’s lifestyle options and choices can generate differences in physical activity and energy expenditure which, over time, lead to health-altering decreases in movement and fitness, and increases in adiposity. While there is agreement that socioeconomic and built environment conditions can promote or inhibit physical activity, there are inconsistencies in the association of built environments and physical activity [24]. These may be due in part to challenges of accurately measuring and recording physical activity [25]. The data from this study suggest (but do not show) that children in urban settings confront physical, cultural, and attitudinal barriers that severely limit physical activity. The urban built environment near the school in this study (i.e. high crime area abutting a major highway) could markedly impede children’s unstructured activity (play). The extremely low levels of PA observed in the study group were particularly concerning in light of ongoing efforts at the school, involving parents and older generation family members, to promote healthy lifestyle changes. Instead, GPS data demonstrated that children were driven for almost all trips to and from school, that they moved little during the school day, and that they spent very little time moving in outdoor recreational facilities such as public parks after school.

The large proportion of students demonstrating little to no amounts of physical activity raises questions about adherence to use of the GPS device. Most of the assessment occurred with the help of the school staff, and while parents were included during consenting procedures, it is possible that parents were not supportive of students wearing GPS devices. Thus if children did not wear their GPS during times of physical activity, “capturing” that activity data could conceivably have been lost. This resulted in a large variability in the GPS measures and, consequently, in low statistical power when correlating the GPS measures with weight, fitness and insulin resistance measures. We set a minimum “threshold” of ninety minutes per day of GPS usage to be included in the activity analysis. While this threshold may be considered somewhat arbitrary, this was done to eliminate data from GPS devices that were unused, or idle, so as not to falsely lower the amount of activity “measured”. Additionally, these data were collected in a single school, with its own distinctive built and cultural environment, and the findings may not be generalizable to other Hispanic communities or other urban communities.

Promoting physical activity during childhood and developing active patterns of moving through one’s daily environment are positive steps toward reducing health consequences associated with obesity and poor fitness [26]. Hispanic youth show unique risks for obesity related illness [3, 27] and, as suggested by the findings of this study of an urban environment, often display extremely low levels of PA which fall far short of federally recommended amounts of physical activity per day. These data provide evidence that reduced movement-associated energy expenditure is one factor contributing to susceptibility for obesity and T2DM risk in this group of children. If environmental interventions designed to increase physical activity can be envisioned and implemented, follow-up studies utilizing GPS will enable accurate assessment of their effect on children’s movement, levels of physical activity, and energy expenditure.

## Conclusion

Utilization of GPS to measure physical activity and its associated energy expenditure revealed that physical activity and EE were extremely low in a group of urban Hispanic children, far below recommendations for health during childhood. Analysis of movement at home, between home and school, and after school showed low levels of physical activity in all settings, indicating that previous school-based measures thus far have not increased PA, and suggesting limited outdoor play options and/or choices near home. This study strengthens the notion that there is need and opportunity for public health interventions that focus on environmental changes in the home, school, and community settings that facilitate daily physical activity for children living in an urban setting. Tools for accurately tracking physical activity are greatly needed. To provide objective documentation of changes in movement associated with and correctly attributable to such interventions, use of GPS technology may be helpful.

## Authors’ information

Supported by the University of Wisconsin-Madison Wisconsin Partnership Program and College of Agricultural and Life Science. The sponsors had no involvement in the design, data collection or analysis.

## Abbreviations

HOMA: Homeostasis model assessment of insulin resistance
PACER: Progressive aerobic cardiovascular endurance run
CVF: Cardiovascular fitness
T2DM: Type 2 diabetes mellitus
GPS: Global positioning system
EE: Energy expenditure
PA: Physical activity.
